# Development of SSR Markers and Assessment of Genetic Diversity in Medicinal *Chrysanthemum morifolium* Cultivars

**DOI:** 10.3389/fgene.2016.00113

**Published:** 2016-06-15

**Authors:** Shangguo Feng, Renfeng He, Jiangjie Lu, Mengying Jiang, Xiaoxia Shen, Yan Jiang, Zhi'an Wang, Huizhong Wang

**Affiliations:** ^1^Zhejiang Provincial Key Laboratory for Genetic Improvement and Quality Control of Medicinal Plants, College of Life and Environmental Sciences, Hangzhou Normal UniversityHangzhou, China; ^2^Institute of Chinese Materia Medica, China Academy of Chinese Medical SciencesHangzhou, China

**Keywords:** *Chrysanthemum morifolium*, EST-SSR, marker development, genetic diversity, phylogenetic relationship

## Abstract

*Chrysanthemum morifolium*, is a well-known flowering plant worldwide, and has a high commercial, floricultural, and medicinal value. In this study, simple-sequence repeat (SSR) markers were generated from EST datasets and were applied to assess the genetic diversity among 32 cultivars. A total of 218 *in silico* SSR loci were identified from 7300 *C. morifolium* ESTs retrieved from GenBank. Of all SSR loci, 61.47% of them (134) were hexa-nucleotide repeats, followed by tri-nucleotide repeats (17.89%), di-nucleotide repeats (12.39%), tetra-nucleotide repeats (4.13%), and penta-nucleotide repeats (4.13%). In this study, 17 novel EST-SSR markers were verified. Along with 38 SSR markers reported previously, 55 *C. morifolium* SSR markers were selected for further genetic diversity analysis. PCR amplification of these EST-SSRs produced 1319 fragments, 1306 of which showed polymorphism. The average polymorphism information content of the SSR primer pairs was 0.972 (0.938–0.993), which showed high genetic diversity among *C. morifolium* cultivars. Based on SSR markers, 32 *C. morifolium* cultivars were separated into two main groups by partitioning of the clusters using the unweighted pair group method with arithmetic mean dendrogram, which was further supported by a principal coordinate analysis plot. Phylogenetic relationship among *C. morifolium* cultivars as revealed by SSR markers was highly consistent with the classification of medicinal *C. morifolium* populations according to their origin and ecological distribution. Our results demonstrated that SSR markers were highly reproducible and informative, and could be used to evaluate genetic diversity and relationships among medicinal *C. morifolium* cultivars.

## Introduction

*Chrysanthemum morifolium* (Ramat.) Kitam is an important member of the family Asteraceae. A large number of *C. morifolium* cultivars are ornamental and medicinally important plants that are planted all over the world (Teixeira da Silva, 2003; Bhattacharya and da Silva, 2006). In addition to their esthetic value, some *C. morifolium* cultivars are used medicinally for their curative effects, particularly for treating common cold, headache, and dizziness (Chinese Pharmmacopoeia Editorial Committee, 2010). Based on growing regions and processing methods, the main domestic varieties of medicinal *C. morifolium* are divided into “Hang-ju,” “Bo-ju,” “Qi-ju,” “Gong-ju,” “Ji-ju,” “Chu-ju,” “Huai-ju,” and “Chuan-ju” (Shao et al., 2010; Zhao et al., 2013). In China, some regions have become important producing areas, such as Tongxiang (Zhejiang Province), Yancheng (Jiangsu Province); Wuzhi (Henan Province), Chuzhou, and Sexian (An'hui Province) (Shao et al., 2010; Wang T. et al., 2013; Chen et al., 2014).

For a long time, the genetic improvement of *C. morifolium* has been impeded because of its genome complexity, high level of heterozygosity, and the occurrence of both inbreeding depression and self-incompatibility (Anderson, 2006). Understanding genetic diversity is very important in plant breeding programs and the conservation of genetic resources. Molecular markers have potentials to reveal the genetic diversity among medicinal *C. morifolium* germplasms. Recently, a few studies have been reported on genetic diversity in cultivated chrysanthemums (Bhattacharya and da Silva, 2006; Xu et al., 2006; Shao et al., 2010; Zhang et al., 2011b), preliminary genetic linkage map construction, and QTL detection (Zhang et al., 2010, 2011a) using RAPD, AFLP, ISSR, and SRAP markers.

Simple-sequence repeats (SSRs), also known as microsatellites, are short tandem repeated motifs that may vary in the number of repeats at a given locus (Tautz, 1989). SSR markers have many advantages over other molecular markers, such as genetic co-dominance. They are multi-allelic, relatively abundant, widely dispersed across the genome, and easily and automatically scored (Powell et al., 1996). Over the past few years, SSR markers have been used in genetic diversity analysis (Dirlewanger et al., 2002; Hasnaoui et al., 2012; Shiferaw et al., 2012; Emanuelli et al., 2013; Ren et al., 2014), parentage assessment (Malysheva et al., 2003), species identification (Shirasawa et al., 2013), and mapping genetic linkage (Temnykh et al., 2000; Olmstead et al., 2008; Lu et al., 2012, 2013). In the genus *Chrysanthemum*, SSR markers have been reported for *C. nankingense* (Wang H. B. et al., 2013). In addition, SSR markers have been used to identify and classify Chinese traditional ornamental chrysanthemum cultivars (Zhang et al., 2014). Nevertheless, current genetic knowledge is very limited for Chinese traditional medicinal chrysanthemum varieties, which hinders genetic conservation and improvement of these endangered, but economically important Chinese medicinal herbs.

In this study, SSR markers were developed and were applied to investigate genetic diversity and phylogenetic relationships among medicinal *C. morifolium* cultivars, with the aim to provide new information that could be used to improve the utilization and conservation of *C. morifolium* genetic resources.

## Materials and methods

### Plant materials and DNA extraction

A total of 32 cultivars of *C. morifolium* were collected from the main distribution areas in China. The sampled germplasms and voucher specimens are shown in Table 1. These varieties were verified and confirmed using the specimens stored in the herbarium at the Institute of Botany, Chinese Academy of Sciences, Beijing, China (http://www.nhpe.org). Voucher samples were deposited in the Zhejiang Provincial Key Laboratory for Genetic Improvement and Quality Control of Medicinal Plants, Hangzhou Normal University, China.

**Table 1:** **List of medicinal ***Chrysanthemum morifolium*** samples included in this study**.

**Original accession**	**Code**	**Voucher number**	**Longitude (E)**	**Latitude (N)**	**Location**
Hangju “Dayangju”	Dyj-1	CM1	120°32′	30°38′	Tongxiang, Zhejiang province
Hangju “Dayangju”	Dyj-12	CM12	120°43′	29°05′	Pan'an, Zhejiang province
Hangju “Dayangju”	Dyj-21	CM21	115°03′	31°18′	Macheng, Hubei province
Hangju “Zaoxiaoyangju”	Zxyj-2	CM2	120°32′	30°38′	Tongxiang, Zhejiang province
Hangju “Zaoxiaoyangju”	Zxyj-13	CM13	120°43′	29°05′	Pan'an, Zhejiang province
Hangju “Xiaoyangju”	Xyj-3	CM3	120°32′	30°38′	Tongxiang, Zhejiang province
Hangju “Xiaoyangju”	Xyj-20	CM20	115°03′	31°18′	Macheng, Hubei province
Hangju “Yizhongdabaiju”	Yzdbj	CM4	120°32′	30°38′	Tongxiang, Zhejiang province
Hangju “Xiaohuangju”	Xhj-5	CM5	120°32′	30°38′	Tongxiang, Zhejiang province
Hangju “Xiaohuangju”	Xhj-10	CM10	120°43′	29°05′	Pan'an, Zhejiang province
Hangju “No. 1 of Jinju”	Jj1	CM6	120°32′	30°38′	Tongxiang, Zhejiang province
Hangju “No. 2 of Jinju”	Jj2-7	CM7	120°32′	30°38′	Tongxiang, Zhejiang province
Hangju “No. 2 of Jinju”	Jj2-14	CM14	120°43′	29°05′	Pan'an, Zhejiang province
Hangju “No. 3 of Jinju”	Jj3	CM8	120°32′	30°38′	Tongxiang, Zhejiang province
Hangju “No. 4 of Jinju”	Jj4	CM9	120°32′	30°38′	Tongxiang, Zhejiang province
Hangju “Chidahuangju”	Cdhj	CM11	120°43′	29°05′	Pan'an, Zhejiang province
Hangju “Dabaiju”	Dbj	CM15	120°25′	33°78′	Sheyang, Jiangsu province
Hangju “Xiaobaiju”	Xbj	CM16	120°25′	33°78′	Sheyang, Jiangsu province
Hangju “Changbanju”	Cbj	CM17	120°25′	33°78′	Sheyang, Jiangsu province
Hangju “Hongxinju”	Hxj	CM18	120°25′	33°78′	Sheyang, Jiangsu province
Hangju “Dahuangju”	Dhj	CM19	120°25′	33°78′	Sheyang, Jiangsu province
Machengju	Mcj	CM22	115°03′	31°18′	Macheng, Hubei province
Boju “Daboju”	Dboj	CM23	115°78′	33°85′	Bozhou, Anhui province
Boju “Xiaoboju”	Xbj	CM24	115°78′	33°85′	Bozhou, Anhui province
Gongju “Zaogongju”	Zgj	CM25	118°43′	29°87′	Shexian, Anhui province
Gongju “Wangongju”	Wgj	CM26	118°43′	29°87′	Shexian, Anhui province
Gongju “Huangyaoju”	Hyj	CM27	118°43′	29°87′	Shexian, Anhui province
Chuju	Cj	CM28	118°32′	32°3′	Chuzhou, Anhui province
Huaiju “Huaixiaohuangju”	Hxhj	CM29	113°38′	35°1′	Wuzhi, Henan province
Huaiju “Huaidabaiju”	Hdbj	CM30	113°38′	35°1′	Wuzhi, Henan province
Huaiju “Huaixiaobaiju”	Hxbj	CM31	113°38′	35°1′	Wuzhi, Henan province
Huaiju “Huaizhenzhuju”	Hzzj	CM32	113°38′	35°1′	Wuzhi, Henan province

Fresh, young leaf tissues from 10 individuals of each cultivar were randomly collected for genomic DNA isolation. The genomic DNA was isolated as described previously (Feng et al., 2013). The integrity and quality of the DNA were evaluated by electrophoresis on 0.8% agarose gels, and the concentration of the genomic DNA samples was determined using a UV spectrometer.

### SSR markers development

A total of 7300 *C. morifolium* EST sequences (total size 3.7 Mb) were retrieved from GenBank (http://www.ncbi.nlm.nih.gov/), with a mean length of 531 bp. These ESTs were analyzed to identify the perfect SSR loci using the MIcroSAtellite (MISA) software (http://pgrc.ipk-gatersleben.de/misa/), following the set to detect tandem repeats of hexa-, penta-, tetra-, tri-, and di-nucleotides, with a minimum number of 4, 4, 5, 7, and 10 tandem arrays of the core repeat, respectively. SSR loci embedded the ESTs with appropriate flanking sequences were selected for primer design using software Primer 3.0 (Untergasser et al., 2012). The parameters for designing the primers were set as follows: primer length with 20 ± 2 nucleotides, amplification product size of 100–300 bp, GC content of 40–60%, and optimum annealing temperature of at least 50°C.

### SSR analysis

A total of 136 SSR primer pairs, targeting at 86 *C. nankingense* (Asteraceae) EST-SSRs (Wang H. B. et al., 2013) and 50 *C. morifolium* EST-SRRs identified in the present study, were synthesized by Shanghai Sangon Biological Engineering Technology and Service Co. Ltd (Shanghai, China). After a trial run of 136 pairs of SSR primers, 55 of them with clearly separated bands, stable amplification, and rich polymorphism were chosen for further analysis.

SSR amplification was performed using 20 μL of PCR mixture solution containing 2 μL 10 × PCR buffer (100 mM Tris-HCl, 100 mM (NH_4_)_2_SO_4_, 100 mM KCl, 1% Triton X-100, pH 8.8), 2 μL MgCl_2_ (20 mM), 0.4 μL dNTPs (10 mM), 1 μL of each primer (10 μM) (forward and reverse), 1 U Taq DNA polymerase (TaKaRa Bio., Kyoto, Japan), and 50 ng genomic DNA templates. PCR amplification was run using a GeneAmp PCR System 9700 (Applied Biosystems, Carlsbad, CA, USA) with the following program: 94°C for 5 min, followed by 35 cycles of 94°C for 40 s, then 40 s at the annealing temperature of each primer pair, 72°C for 1 min 30 s, and a final extension at 72°C for 10 min. PCR products were separated on 1.5% agarose gel, stained with ethidium bromide and photographed under UV light. Sanger sequencing was used to confirm SSRs in amplified genomic DNA fragments as described previously (Lu et al., 2013).

### Data analysis

Only reproducible and consistent SSR fragments were scored as present (1) or absent (0) for each of the SSR markers. The polymorphism information content (PIC) of each pair of SSR primers was calculated using the formula:

$$PIC=1-\left(\sum_{i\;=\;1}^{\text{n}}p_{i}^{2}\right)-\left(\sum_{i\;=\;1}^{n-1}\sum_{j\;=\;i+1}^{n}2q_{i}^{2}q_{j}^{2}\right)$$

Where *n* is the number of alleles (marker), *q*_*i*_ is the ith allele frequency, and *q*_*j*_ is the jth allele frequency (Botstein et al., 1980), The cluster analysis was conducted by NTSYS-pc version 2.10e software (Rohlf, 2000). A dendrogram was constructed using the unweighted pair group method with an arithmetic mean (UPGMA) based on similarity matrices calculated using the simple matching (SM) coefficient (Nei and Li, 1979). The data was also analyzed using principal coordinate analysis (PCoA) (Gower, 1966) to further demonstrate the multiple dimensional distributions of the chrysanthemum cultivars in a scatter-plot.

## Results

### SSR markers development and primer design

In total, 218 microsatellites were detected in 207 ESTs (Tables 2, 3). Among them, 10 (4.83%) ESTs contained more than one SSR loci (Table 2). Information about 218 SSR loci was showed in Supplementary Material. Of all detected SSR loci, hexa-nucleotide repeats were the most abundant with 134 loci, (61.47% of the total), followed by tri-nucleotide repeats with 39 loci (17.89% of the total), di-nucleotide repeats with 27 loci (12.39% of the total), tetra-nucleotide repeats with 9 loci (4.13% of the total), and penta-nucleotide repeats with 9 loci (4.13% of total) (Tables 2, 3). After removal of those ESTs with too short or inappropriate flanking sequences for primer design, 50 EST-SSRs were selected for primer design (Table 4).

**Table 2:** **Characterization of EST-SSRs in ***C. morifolium*** genome**.

**Parameter**	**Value**
Total number of ESTs searched	7300
Total size of examined sequences (bp):	3,717,958
Total number of ESTs with SSRs	207
Total number of ESTs with a single SSR	197
Total number of ESTs with more than 1 SSR	10
Repeat types	
Di-nucleotide	27 (12.39%)
Tri-nucleotide	39 (17.89%)
Tetra-nucleotide	9 (4.13%)
Penta-nucleotide	9 (4.13%)
Hexa-nucleotide	134 (61.47%)
Total number of SSRs identified	218

**Table 3:** **Distributions of microsatellite motifs observed in ***C. morifolium*** ESTs in the GenBank database**.

**SSR motif**	**Number of repeat units in ESTs**								
	**3**	**4**	**5**	**6**	**7**	**8**	**9**	**≥10**	**Total**
Di-nucleotide	–	–	–	–	–	–	–	27	27
(AC)n	–	–	–	–	–	–	–	10	10
(AG)n	–	–	–	–	–	–	–	13	13
(AT)n	–	–	–	–	–	–	–	4	4
Tri-nucleotide	–	–	–	–	19	12	5	3	39
(AAC)n	–	–	–	–	4	4	3	–	11
(AAG)n	–	–	–	–		2	–	–	2
(AAT)n	–	–	–	–	3	2	–	2	7
(ACC)n	–	–	–	–	8	2	1	1	12
(ACT)n	–	–	–	–	1			–	1
(AGC)n	–	–	–	–		1	1	–	2
(ATC)n	–	–	–	–	3	1		–	4
Tetra-nucleotide			6	2		1		–	9
Penta-nucleotide		9						–	9
Hexa-nucleotide	110	17	6	1				–	134
Total	110	26	12	3	19	13	5	30	218

**Table 4:** **Polymorphism of 55 SSR primer pairs in medicinal ***Chrysanthemum morifolium*** samples**.

**Primer pair (ID)**	**Primer Sequences (5′-3′)**	**Repeat motif**	**T_m_**	**Size**	**No. of loci**	**No. of Polymorphic loci**	**Polymorphic loci %**	**PIC**	**References**
CMeSSR001	F:ATTCTGTCACTCAAACACCAC	(CATC)_8_	55.3	251	26	26	100.00	0.978	This study
	R:GGTTCAAACGAGCTAAATTACA								
CMeSSR002	F:CTCACCATTTTCAGACCATTAT	(ACA)_7_	55.4	251	26	26	100.00	0.976	This study
	R:ACACATCTTGTACCTCTTGGTT								
CMeSSR003	F:CTTTTTCACACACACTCAACAT	(TAA)_7_	54.5	256	26	26	100.00	0.976	This study
	R:TTGGAGACGTTGTTGTAAAGTA								
CMeSSR004	F:AAAATGTTAGGTGCAGGATTAC	(AAT)_7_	54.3	248	27	27	100.00	0.977	This study
	R:AAAAACCGTTCCAGATTACAC								
CMeSSR005	F:AAAACCTTCACTAGATCACACC	(CAC)_7_	56.3	252	29	29	100.00	0.978	This study
	R:TTTCAGTATCTTGGACCAGTCT								
CMeSSR006	F:ATTCTCTTAATTAGCCAGCAAG	(CAC)_7_	54.3	247	17	17	100.00	0.958	This study
	R;GTGAATCGTAAATTCAGTTGG								
CMeSSR007	F:GTCCTCCTTCAAAGCAAA	(CTA)_7_	51.3	157	28	28	100.00	0.976	This study
	R:GACGATTAATTATTGGGTAATA								
CMeSSR009	F:AGTGATGATGAATTGAAAGAGC	(AAT)_8_	56.4	258	18	17	94.44	0.959	This study
	R:CTCTCAAGTGTTGAAGGAACTC								
CMeSSR010	F:CATTTTCTTCATGGTACTCACA	(CAC)7	56.4	169	27	27	100.00	0.978	This study
	R:GTGAGGATGGAAATCTAGTAGG								
CMeSSR011	F:AGGACAACTCAACTGTTAGGAG	(CCA)_7_	57.2	255	20	19	95.00	0.964	This study
	R:GTTTCTCAACCTCTTCTTCATC								
CMeSSR012	F:ATTCCCAACCTTCTTTAACC	(CA)_11_	54.1	254	29	29	100.00	0.979	This study
	R:AACTAAATCACCATCTCTTGCT								
CMeSSR013	F:ATGAGAGGGAAATAGAAAGTGA	(GTAATA)_3_(TAA)_4_	56.4	219	26	26	100.00	0.973	This study
	R:TACTTGACGCTAACGGAGTAGT								
CMeSSR014	F:CAAAACTTTCAACAGAGTCATC	(CAACAT)_3_(CAG)_4_	54.5	281	35	35	100.00	0.983	This study
	R:AGAAATAACGACTGGTCAGATT								
CMeSSR015	F:TCTTGGTCAGCTTAATTACTCA	(TGG)_7_…(TGG)_4_(AGG)_4_	57.1	236	30	29	96.67	0.979	This study
	R:CATCACCTCCTCCTCCTC								
CMeSSR016	F:GAATACTAAATGGGTGGAAGAA	(GGA)_4_(GGT)_4_	55.3	250	18	18	100.00	0.967	This study
	R:GCAAATAGATGTCCTTTAGGG								
CMeSSR017	F:TCATGAAATCCGTGTATATGTC	(AC)_5_a(AC)_6_	54.5	229	30	30	100.00	0.980	This study
	R:ACCCTAATTCTCAAAATGAACC								
CMeSSR018	F:ATCTACTATCCAAGCCATGAAC	(CAC)_5_…(GGT)_10_…(TGG)_6_	56.8	264	25	23	92.00	0.973	This study
	R:TATCCACCACCACCACCA								
gi298295865	F:ACTCACTTGCCCCATTTGTC	(AACCCT)_5_	59.8	146	15	15	100.00	0.957	Wang H. B. et al., 2013
	R:AGAGAAGCTCTCCAGGGACC								
gi298300528	F:AGGGCATCGATAATCCATCA	(ATATC)_4_	56.8	135	12	12	100.00	0.938	Wang H. B. et al., 2013
	R:AGATACGTGCCCATTTGAGG								
gi298295793	F:ATAGAATTCCCCGACGACAA	(CCCTAT)_4_	56.8	111	15	14	93.33	0.948	Wang H. B. et al., 2013
	R:GGCGGTTGAGATTGATAGGA								
gi298296818	F:ATGTCCAGCTTGATGGGAAG	(GTG)_7_	58.8	210	24	23	95.83	0.977	Wang H. B. et al., 2013
	R:GGCCCCTTGCAAATCCTC								
gi298298301	F:CTTGACCGAAACACCGAAAT	(TTG)_9_	56.8	198	17	16	94.12	0.958	Wang H. B. et al., 2013
	R:TGGCATCCTAGTTAGCAGCA								
gi298299323	F:GCACATTTCCTTCATGGGTT	(ACA)_9_	57.8	264	33	33	100.00	0.982	Wang H. B. et al., 2013
	R:TCCACGGTTTCAGATGATGA								
gi298297301	F:TCAAACACCACCACCAACAC	(CATC)_8_	58.8	167	25	25	100.00	0.973	Wang H. B. et al., 2013
	R:ATGTCACCAAGTCCTGGTCC								
51	F:CCCCCTCTTCTTCTTCAACC	(CCAA)_4_	57.8	202	22	22	100.00	0.974	Wang H. B. et al., 2013
	R:CAATAGAAAGCGCGTGACAA								
53	F:TCGAAGACAATCAGCACCTG	(ATG)_7_	57.8	233	18	17	94.44	0.961	Wang H. B. et al., 2013
	R:TAAGTGTTCTTCCAGCGCCT								
64	F:GGCGATGGATGATGATGATT	(TTC)_9_	56.8	267	22	22	100.00	0.991	Wang H. B. et al., 2013
	R:GAAAGAGGTGGATCGGATGA								
86	F:AAACCACCAAACCCATCAAA	(TGG)_8_	54.7	223	25	25	100.00	0.990	Wang H. B. et al., 2013
	R:AACTTTGCCAGCATCGACTT								
135	F:CATTCCTACCCATCCCTCCT	(GTGGAG)_4_	58.8	100	31	31	100.00	0.993	Wang H. B. et al., 2013
	R:CGCATGAGTGAGCCTAATGA								
204	F:TGAGCTTCATCCGCTTCTTT	(TGA)_8_	55.8	262	10	10	100.00	0.985	Wang H. B. et al., 2013
	R:TGGTCGTATTCCGTCCATTT								
219	F:AAAAGGTTGTGAGTGGGTCG	(GGGAAG)_4_…(TGAGGG)_4_	57.8	228	26	25	96.15	0.976	Wang H. B. et al., 2013
	R:CCTCGGTCGATAAATCTCCA								
221	F:AACCATGAATCCAGACACCC	(TCA)_7_	57.8	181	20	20	100.00	0.964	Wang H. B. et al., 2013
	R:ACCAAGCCAGTCGAGTTTTG								
235	F:GCCCCAATTTATTCACTCCA	(AAC)_6_	57.8	257	30	30	100.00	0.979	Wang H. B. et al., 2013
	R:GCTCTTCCTCGTAAGCATCG								
262	F:TCTGCCAGCTTTGGGTAACT	(CTTTTT)_4_	57.8	260	13	13	100.00	0.946	Wang H. B. et al., 2013
	R:GTGCGCCTGTATTGACTTGA								
270	F:AGGTGGAAAATACTGTGCGG	(ATAGTA)_4_	57.8	139	19	19	100.00	0.966	Wang H. B. et al., 2013
	R:TGTTTCTGCACCTCAACAGC								
285	F:CCGGTGTTCGGTATAAATGG	(GTG)_7_	56.8	116	11	11	100.00	0.965	Wang H. B. et al., 2013
	R:ACAATTCGCTTCGGCTCTAA								
312	F:GGCCCAAGTTTGAGACAAAA	(AAG)_7_	56.8	219	36	36	100.00	0.985	Wang H. B. et al., 2013
	R:TCGGTATAAGTGCACCACGA								
313	F:GGCGTTCTCTTCCATTTCAA	(GAA)_7_	56.8	253	37	37	100.00	0.984	Wang H. B. et al., 2013
	R:GTTTTGGACCTTGCTTCTGC								
320	F:GGTCCTTCGTTTCATTTGGA	(TGG)_7_	57.8	235	21	21	100.00	0.973	Wang H. B. et al., 2013
	R:CGGGGGTAGGAATAGAAAGC								
327	F:GAATGCAGCCTCAACAACAA	(TCAAAG)_4_	55.8	219	27	27	100.00	0.979	Wang H. B. et al., 2013
	R:GAGCCGCCATTGTCATATTT								
357	F:ACCCAACCTGAACAAGATGC	(GGGTCA)_4_	58.8	252	24	24	100.00	0,990	Wang H. B. et al., 2013
	R:ATACTGCTGCCACTGACCCT								
581	F:CCAATCCCAAACACTCCCTA	(CA)_16_	58.8	223	24	24	100.00	0.976	Wang H. B. et al., 2013
	R:GCCGTTACCACTGCTCTTTC								
984	F:TCAAAACCCATCATCACCCT	(ACA)_7_	55.8	186	28	27	96.43	0.979	Wang H. B. et al., 2013
	R:CGGCGTTTGTATCTTGGTTT								
995	F:TTGTTCCACGTGACGAGATT	(TGTTGG)_4_	56.9	244	16	16	100.00	0.961	Wang H. B. et al., 2013
	R:CTCCCAAATGACCCATCATC								
1036	F:CTTTGGTAAGCGAAGGCTGT	(AATG)_7_	55.7	153	16	16	100.00	0.955	Wang H. B. et al., 2013
	R:GCCATTTGTAAGCGGTTTGT								
1187	F:GAAAGCGATCATTGGGAAAA	(GAGAAG)_4_	54.7	112	19	19	100.00	0.963	Wang H. B. et al., 2013
	R:TTACCCGTACATTCGGGATT								
1424	F:TAAAATCCATCCGTCCATCC	(CAA)_7_	57.8	278	26	26	100.00	0.975	Wang H. B. et al., 2013
	R:CTTCCATATCTGCCAGTGGG								
1428	F:AACGCCCAAAACACCAACT	(AC)_9_	56.6	224	26	26	100.00	0.978	Wang H. B. et al., 2013
	R:TAGAACCTTGTGCCCCCATA								
1520	F:AAATCACGGATCCCCTTCTT	(ATAGA)_4_	57.8	158	32	32	100.00	0.982	Wang H. B. et al., 2013
	R:TTATCATCTTGGGGAGTGGC								
1584	F:CCTCCTCAAAACGACCATGT	(ACA)_7_	57.8	263	24	23	95.83	0.973	Wang H. B. et al., 2013
	R:CGTCCCCATTACAATATCCG								
1742	F:AAGTGATAAGATGGGTGGCG	(TGG)_7_	57.8	166	28	28	100.00	0.974	Wang H. B. et al., 2013
	R:GGTGGAGGCTCATTCAAATC								
1762	F:GCGTCAAATTACTGGTGGCT	(AAC)_6_	56.8	259	22	21	95.45	0.972	Wang H. B. et al., 2013
	R:GTCTCATTTTCCGGCGATAA								
1773	F:CAAATGGGGTCGTTACGAAT	(CAT)_6_	55.8	215	17	17	100.00	0.965	Wang H. B. et al., 2013
	R:AATCCCCGAATTCCCAATAG								
1774	F:TCACCACCACCACTGTCACT	(CACCGG)_4_	59.8	276	38	38	100.00	0.984	Wang H. B. et al., 2013
	R:TGTGGGCTCTAGAGGTTTGG								
1779	F:AAAGTCCCCTTGCTTGTTCC	(AACC)_5_	57.8	148	25	25	100.00	0.975	Wang H. B. et al., 2013
	R:CGACTCCATTTGATCCACCT								
Total					1319	1306			
Average					23.98	23.75	98.90%	0.972	

A total of 136 SSR primer pairs, including 50 *C. morifolium* EST-SSRs identified above and 86 *C. nankingense* EST-SSRs (Wang H. B. et al., 2013), were screened using three genomic DNA samples. Fifty-five of the primer pairs (40.44%) generated reproducible polymorphic DNA amplification products. The amplified bands with clear and expected size were sequenced. The corresponding repeat motifs were validated for 50 EST loci by Sanger sequencing. Finally, 17 novel *C. morifolium* EST-SSRs were developed successfully, and 38 *C. nankingense* EST-SSRs were confirmed with transferability for application in a related species. These 55 pairs of SSR primers were used for further genetic diversity analysis in *C. morifolium* cultivars (Table 4).

### SSR analysis

**Figure 1:**
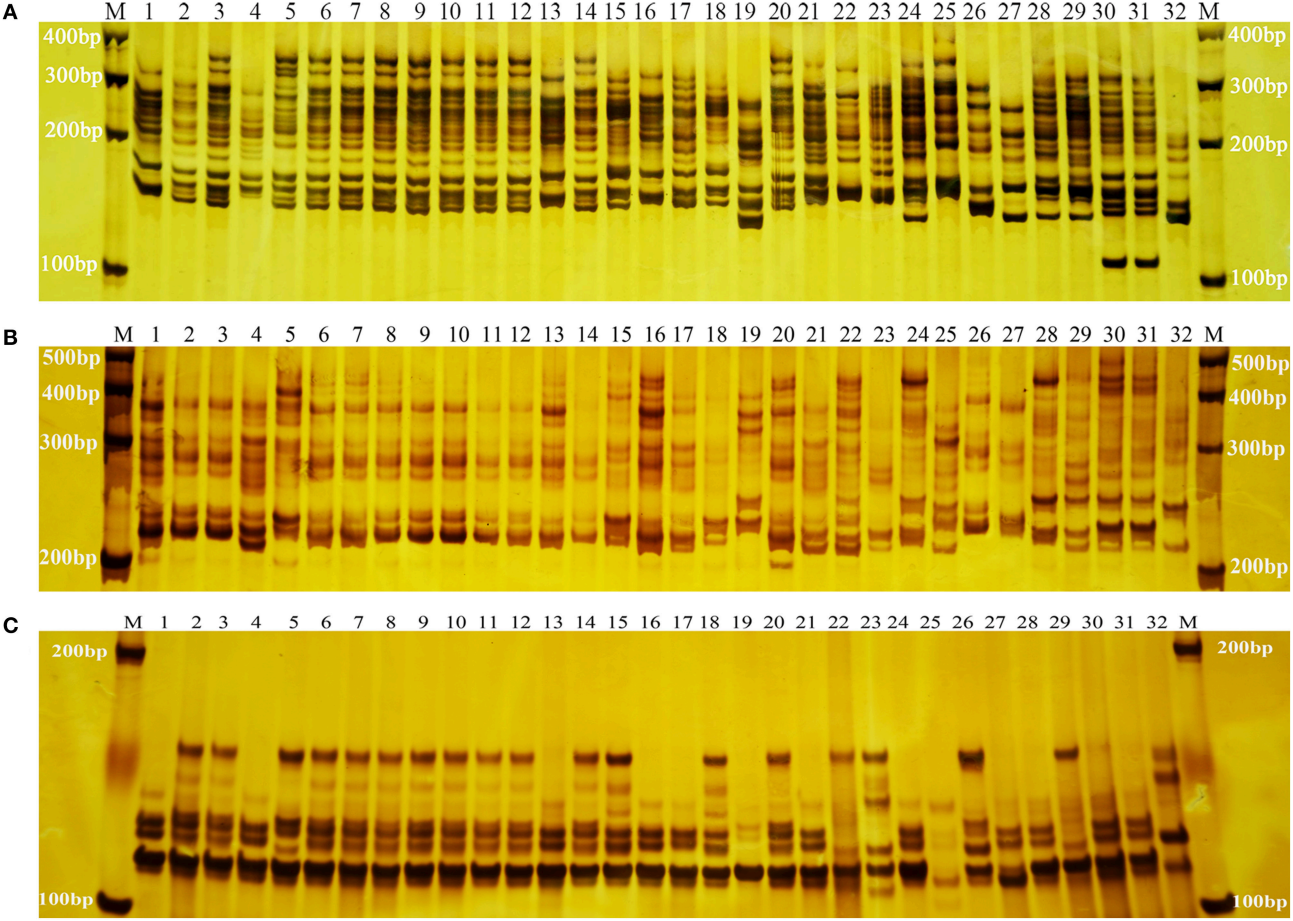
**SSR amplification profiles of primer pairs CMeSSR001 (A), 219 (B), and 285 (C)**. *Lane M*: DNA molecular standards with length (bp) on left and right. *Lanes 1–32*: genotypes of the 32 *Chrysanthemum morifolium* samples (CM1–CM32) in Table 1.

The 55 SSR primer pairs generated a total of 1319 fragments with an average of 23.98 fragments per primer pair and a range of 10 (primer pair ID. 204) to 38 (primer pair ID. 1774) (Table 4). A total of 1306 were polymorphic. The percentage of polymorphic bands across the primer pairs varied from 92.00 to 100.00% (Table 4), with an average 98.90%. Three representative profiles (primer pair ID. CMeSSR001, 219 and 285) are shown in (Figures 1A–C). The PIC value varied from 0.938 to 0.993 with an average of 0.972 (PIC > 0.5), which indicated that these loci contained a considerable amount of genetic information that could be used in genetic diversity studies on Chrysanthemum germplasms.

### Genetic diversity and relationships among genotypes

A total of 1319 loci were accounted to calculate the genetic diversity among the 32 Chrysanthemum cultivars. Binary data matrices produced by SSRs were used to estimate the genetic similarity of the genotyped Chrysanthemum samples. The pairwise similarity coefficient among the 32 cultivars ranged from a maximum of 0.809 (between Huaiju “Hdbj” and Huaiju “Hxbj”) to a minimum of 0.533 (between Huangju “Jj3” and Gongju “Wgj”).

**Figure 2:**
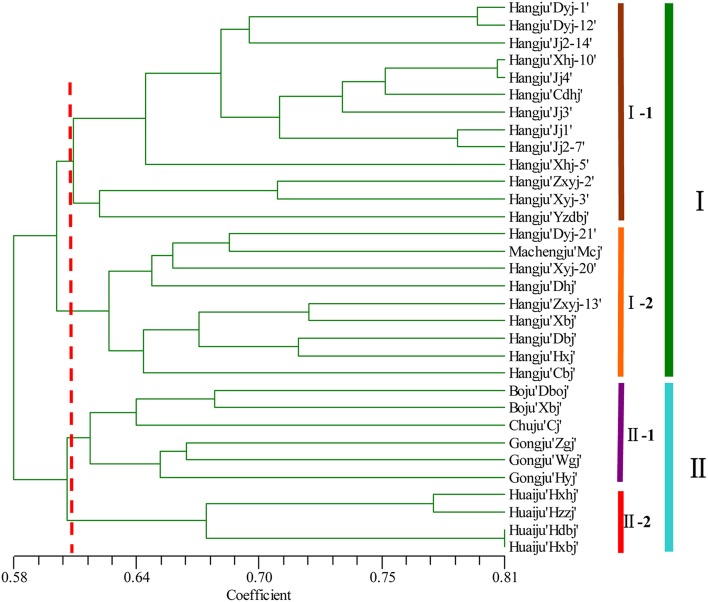
**Relationships among ***Chrysanthemum morifolium*** varieties based on the genetic similarities between DNA fingerprinting patterns from SSR markers used in the UPGMA dendrogram**.

A dendrogram using UPGMA analysis was constructed based on the corresponding genetic similarity coefficient among the tested 32 *C. morifolium* populations (Figure 2). In this study, all the *C. morifolium* samples could be grouped into two main clusters, with a similarity index of 0.584. Cluster I consisted of 22 cultivars, including all the “Hangju” and “Machengju” samples. This cluster was further subdivided into three subgroups. Subgroup “I-1” consisted of 13 samples, all of which belong to Hangju cultivars. Machengju “Mcj” and eight Hangju cultivars were assigned to subgroup “I-2.” Group II comprised of 10 cultivars, which belonged to “Boju,” “Huaiju,” “Chuju,” and “Gongju.” Among them, the “Boju,” “Chuju,” and “Gongju” cultivars were classified into subgroup II-1, while four “Huaiju” cultivars constituted subgroup II-2.

### Principal coordinate analysis

**Figure 3:**
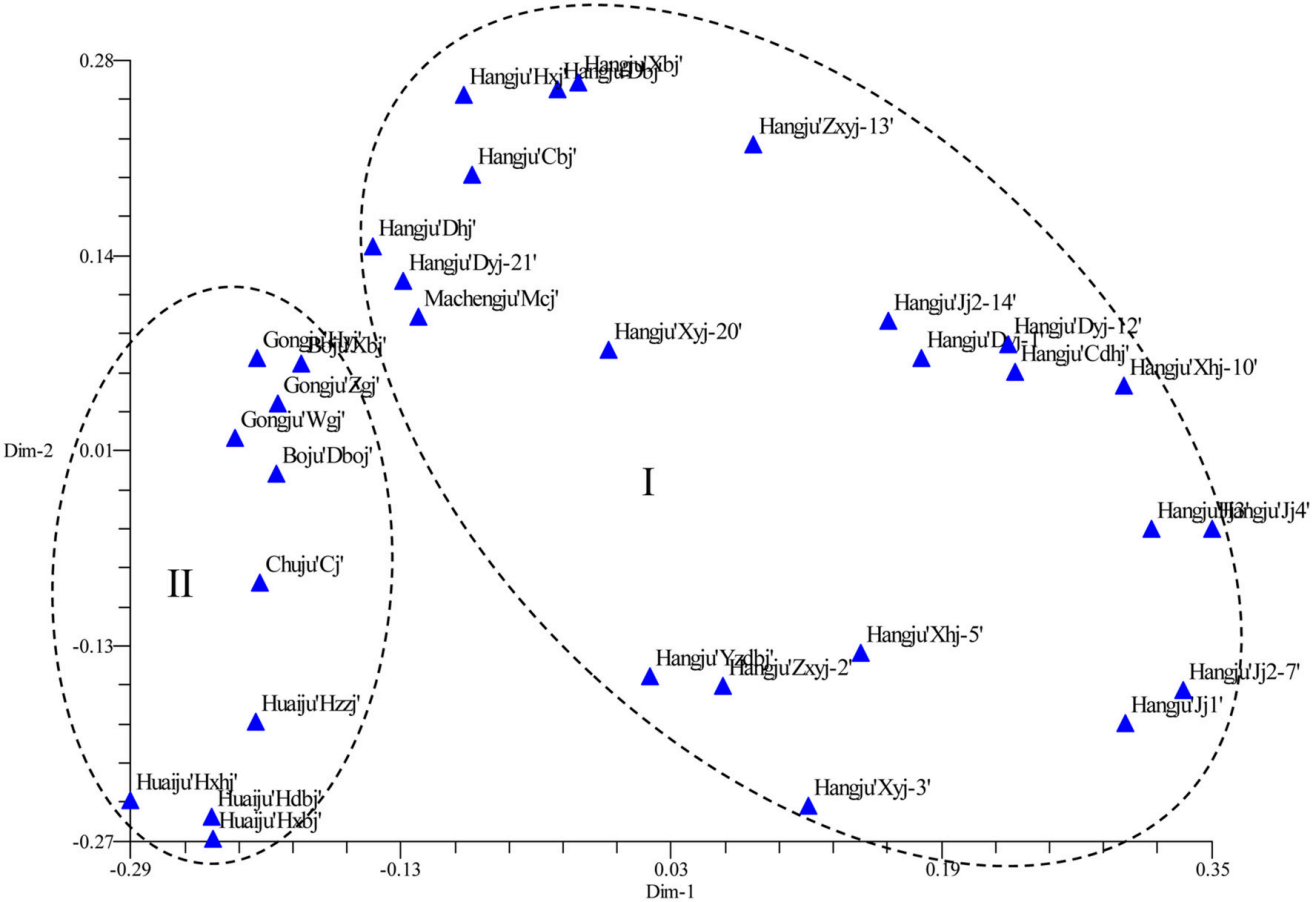
**Two-dimensional projection of the PCoA of 32 ***Chrysanthemum morifolium*** samples based on SSR markers along the first two principal axes**.

The SSR data were subjected to PCoA in order to obtain an alternative view of the phylogenetic relationships among the cultivars (Figure 3). In the two-dimensional PCoA plot, *C. morifolium* cultivars were divided into two groups (Figure 3), which was similar to the pattern as shown by the UPGMA dendrogram. The first two principal axes explained 10.60 and 6.70% of the total molecular variation observed, respectively.

## Discussion

Compared with anonymous markers, SSR markers, as a type of co-dominance markers, may yield more accurate estimates of genetic diversity. SSRs have been used successfully to determine genetic diversity among many plants (Dirlewanger et al., 2002; Hasnaoui et al., 2012; Shiferaw et al., 2012; Emanuelli et al., 2013; Ren et al., 2014). SSRs were previously identified in *C. nankingense* and *C. nankingense* SSRs were proved to be useful for genetic analysis in the genus *Chrysanthemum* and its related genera (Wang H. B. et al., 2013). In the present study, we found that 44% (38 out 86) of *C. nankingense* SSRs were also proved to be useful for genetic diversity study among medicinal *C. morifolium* cultivars (Table 4).

A previous study used 20 SSR markers for identification and classification of Chinese traditional ornamental Chrysanthemum cultivars (Zhang et al., 2014). However, few studies have explored development and application of SSR markers for genetic diversity among medicinal *C. morifolium* cultivars. The diversity and genetic relationship among 29 *C. morifolium* populations were investigated using the types of dominant molecular markers (Shao et al., 2010). The present study report discovery of novel SSRs in *C. morifolium*.

The SSR markers selected in this study yielded reproducible polymorphic bands in 32 *C. morifolium* cultivars and showed that they provide a powerful and reliable molecular tool for analyzing genetic diversity and relationships among *C. morifolium* cultivars. In this study, 98.90% of the bands generated by the SSR assay were polymorphic, which was higher than the polymorphic proportions of 53.85% detected by SSR among celery cultivars (Fu et al., 2014), 97.40% among grass pea from different regions (Shiferaw et al., 2012), and 97.50% among melon accessions (Kacar et al., 2012). Molecular markers with higher PIC values have a greater ability to identify cultivars. A locus with a PIC greater than 0.5 is considered to be highly diverse, as a previous study reported (Botstein et al., 1980). The PIC values of the SSR markers used in the Chrysanthemum cultivars analysis ranged between 0.938 and 0.993, with an average of 0.972, which indicated that the highly informative SSR markers could be employed in genetic diversity studies of medicinal Chrysanthemum cultivars.

Evaluation of genetic diversity and relationship among plant populations is the foundation of selective breeding programs. Using SSR markers, our study found considerable diversity among Chrysanthemum cultivars, which could be used in breeding programs for Chrysanthemum improvement. According to their origin and ecological distribution, 32 *C. morifolium* cultivars were classified into six sources: Hangju, Machengju, Chuju, Boju, Gongju, and Huaiju (Table 1). A dendrogram constructed with SSR data using the UPGMA method indicated that the *C. morifolium* cultivars were grouped into two main groups. All the Hangju cultivars were clustered in the first group along with Machengju “Mcj” (Group I), which means that the genetic relationship between Hangju cultivars and Machengju “Mcj” is very close, consistant with a previous study (Shao et al., 2010). The main growing regions for Boju, Gongju and Chuju are Bozhou (115°78′, 33°85′), Shexian (118°43′, 29°87′), and Chuzhou (118°32′, 32°3′) in Anhui Province, China. In theory, the genetic relationships between the cultivars of these three *C. morifolium* populations may be closer than between other *C. morifolium* populations (Hangju, Machengju and Huaiju). In our study, all the Boju, Chuju and Gongju cultivars were grouped together within subgroup II-1, which confirmed the inference above (Figures 2, 3). The Huaiju cultivars collected from Wuzhi, Henan Province (113°38′, 35°1′), were grouped into subgroup II-2. Geographically, Henan Province is adjacent to Anhui Province, which may explain why the four Huaiju cultivars have a close relationship with other three *C. morifolium* populations (Chuju, Boju, and Gongju) (Figure 2). The results of the present study showed that cluster analysis using SSR markers mainly supported the classification of medical *C. morifolium* accessions according to their origin and ecological distribution.

Increased urbanization has meant that *C. morifolium*' cultivation has greatly declined and some populations are rare. Therefore, it is imperative to undertake effective measures to protect *C. morifolium* germplasms. Our study found that there was a high level of genetic diversity between Chrysanthemum populations. As the study by Shao et al. (2010) suggested, a priority for *in situ* conservation should be to rescue and conserve the core populations.

In conclusion, our study demonstrates that SSR technology is a powerful tool for evaluating genetic diversity and relationships among the medical *C. morifolium* cultivars. SSR analysis showed that medicinal *C. morifolium* populations could be classified according to their origin and ecological distribution. In future studies, more medicinal *C. morifolium* cultivars will be included to verify whether these findings are true for more closely related taxa.

## Author contributions

Conceived and designed the study: SF, HW. Collected plant samples XS, ZW. Performed the experiments: SF, RH, MJ. Analyzed the data: SF, RH, JL, YJ. Wrote the manuscript: SF, HW.

## Funding

This study was supported in part by the Zhejiang Provincial Natural Science Foundation of China (LQ13H280006); the National science and technology support Programme project (2011BAI04B02); Zhejiang Provincial New Agricultural Varieties Breeding Of Traditional Chinese Medicinal Materials Major R&D Projects (2012C12912).

### Conflict of interest statement

The authors declare that the research was conducted in the absence of any commercial or financial relationships that could be construed as a potential conflict of interest.
